# Association Between Ischemic Stroke and COVID-19 in China: A Population-Based Retrospective Study

**DOI:** 10.3389/fmed.2021.792487

**Published:** 2022-02-21

**Authors:** Minghuan Wang, Han Zhang, Yuqin He, Chuan Qin, Xingyuan Liu, Mingqian Liu, Yuhong Tang, Xiaohua Li, Guang Yang, Yingxin Tang, Gang Liang, Shabei Xu, Wei Wang

**Affiliations:** ^1^Department of Neurology, Tongji Medical College, Tongji Hospital, Huazhong University of Science and Technology, Wuhan, China; ^2^Information Center, Wuhan Municipal Health Commission, Wuhan, China; ^3^Winning Health Technology Group Co. Ltd, Shanghai, China

**Keywords:** COVID-19, risk factors, prior stroke, acute ischemic stroke, MRS

## Abstract

**Background and Purpose:**

To investigate the effect of prior ischemic stroke on the outcomes of patients hospitalized with coronavirus disease 2019 (COVID-19), and to describe the incidence, clinical features, and risk factors of acute ischemic stroke (AIS) following COVID-19.

**Methods:**

In this population-based retrospective study, we included all the hospitalized positive patients with COVID-19 at Wuhan City from December 29, 2019 to April 15, 2020. Clinical data were extracted from administrative datasets coordinated by the Wuhan Health Commission. The propensity score matching and multivariate logistic regression analyses were used to adjust the confounding factors.

**Results:**

There are 36,358 patients in the final cohort, in which 1,160 (3.2%) had a prior stroke. After adjusting for available baseline characteristics, patients with prior stroke had a higher proportion of severe and critical illness and mortality. We found for the first time that the premorbid modified Rankin Scale (MRS) grouping (odds ratio [*OR*] = 1.796 [95% *CI* 1.334–2.435], *p* < 0.001) and older age (*OR* = 1.905 [95% *CI* 1.211–3.046], *p* = 0.006) imparted increased risk of death. AIS following COVID-19 occurred in 124 (0.34%) cases, and patients with prior stroke had a much higher incidence of AIS (3.4%). Logistic regression analyses confirmed an association between the severity of COVID-19 with the incidence of AIS. COVID-19 patients with AIS had a significantly higher mortality compared with COVID-19 patients without stroke and AIS patients without COVID-19.

**Conclusions:**

Coronavirus disease 2019 patients with prior stroke, especially those with the higher premorbid MRS or aged, have worse clinical outcomes. Furthermore, COVID-19 increases the incidence of AIS, and the incidence is positively associated with the severity of COVID-19.

## Introduction

At present, with more than 80 million survivors (1), the global burden of ischemic stroke (IS) is high (2). Prior IS is a suggested risk factor contributing to the severity and mortality of coronavirus disease 2019 (COVID-19) (3, 4), but this association is indicated by studies of limited sample size (5). Patients with prior stroke are particularly vulnerable toward pulmonary and inflammatory complications due to their frequent disability (6), greater age, and higher prevalence of smoking, hypertension, and cardiovascular disease, all of which were important predictors of poor COVID-19 outcomes (7). As such, patients with prior stroke are a special population, and their specific risk factors for severe disease and death after COVID-19 infection need to be further studied.

Moreover, a temporal relationship between various respiratory viral infections and increased risk of stroke has been reported (8), and COVID-19 had been reported to increase the incidence of acute ischemic stroke (AIS) in several case series and meta-analyses (9, 10). However, the reported incidence of AIS varied widely between studies (11), and the predictors, clinical characteristics, and outcomes of AIS in patients with COVID-19 need further study.

Wuhan city is the capital of Hubei province in China, which emerged as the original epicenter of COVID-19, with some 50,000 individuals infected and nearly 4,000 deaths recorded during the early phase of this pandemic. Having access to detailed medical records for the entire cohort, we undertook to investigate the effect of prior stroke on the severity of COVID-19 and the risk of in-hospital death among hospitalized patients. We furthermore describe the incidence, clinical features, risk factors, and outcomes of patients with AIS following their COVID-19 hospitalization.

## Methods

### Study Design and Participants

**Figure 1:**
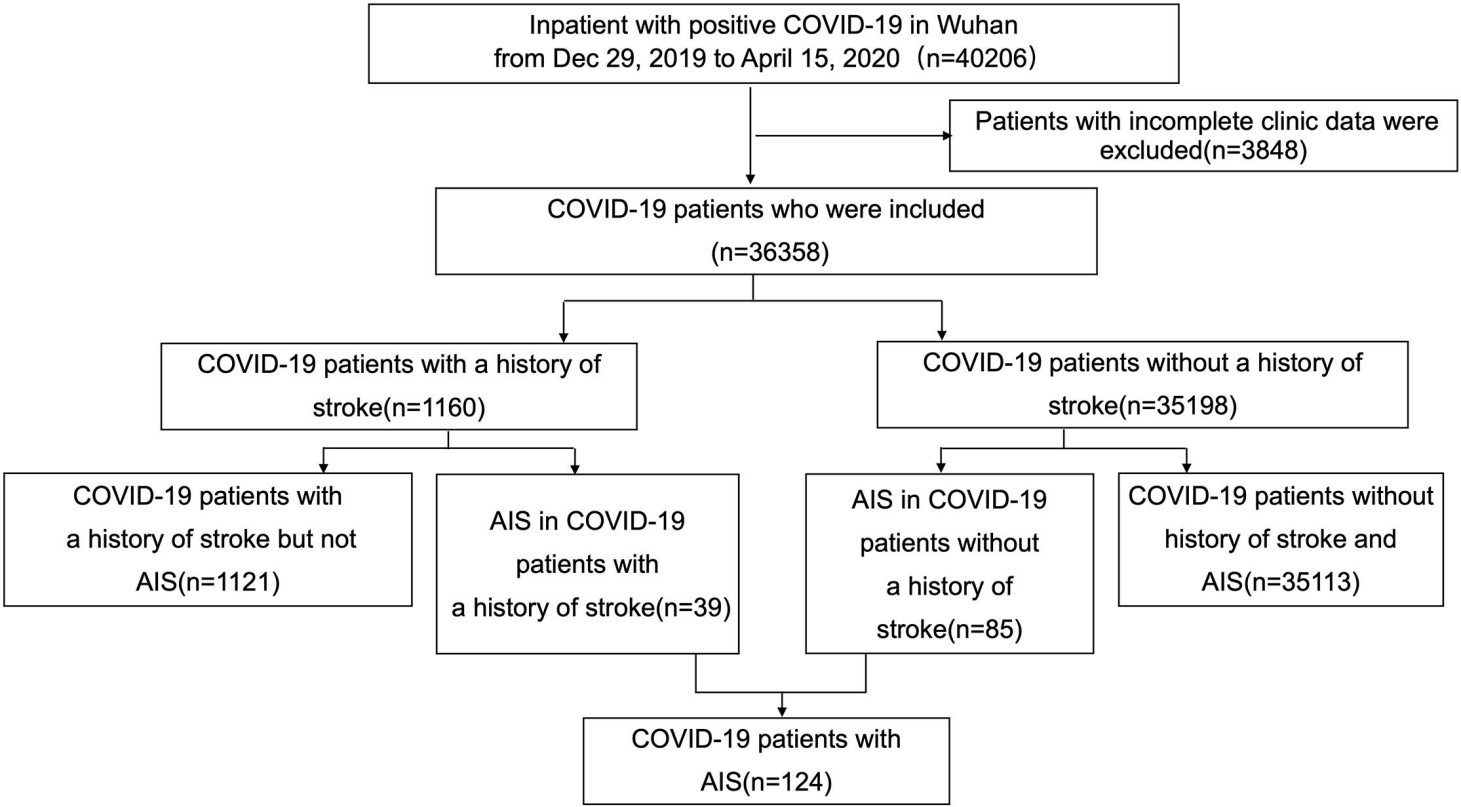
Flowchart of patient inclusion and exclusion.

This is a retrospective cohort study using data collected from all inpatients in Wuhan with positive COVID-19 test results recorded from Dec 29, 2019, when patients were admitted to hospital for the first time, and extending to April 15, 2020, which was the first day with no new cases declared in Wuhan city. The compilation of data was coordinated by the Wuhan Health Commission, which mandated the reporting of clinical information from every designated hospital in Wuhan that had admitted patients with laboratory confirmed COVID-19 (Figure 1). Patient follow-up continued through July 1, 2020. This study was designed by the authors and approved with provision of a waiver of authorization and informed consent by the Ethics Committee of Tongji Hospital (IRB ID: TJ-C20200121).

### Data Collection and Data Extraction

The demographic, clinical, laboratory, treatment, and outcome data were extracted from electronic medical records of 61 Wuhan hospitals and mobile cabin hospitals using a standardized data collection and processing method (detail in Supplementary Materials). The laboratory data were collected upon patient admission. Systolic blood pressure (SBP) was recorded on admission. We excluded patient records with incomplete clinic data. All data were checked by two physicians (HZ and YH), and a third researcher (MW) adjudicated any difference in the interpretation between the two primary reviewers. Patients with COVID-19 were divided into four categories according to the severity as Mild, Moderate, Severe, and Critical according to Diagnosis and Treatment Protocol for Novel Coronavirus Pneumonia (Trial Version 6) released by the National Health Commission (detail in Supplementary Materials). Prior stroke was determined according to the electronic medical record. The COVID-19-associated AIS was defined as the AIS that occurred during hospitalization of patients with COVID-19 or COVID-19 was found at the same time as the hospital visit due to AIS. We excluded patients who were infected after the onset of AIS. The diagnosis of AIS required the validation by brain CT or MRI. Neurological recovery in patients with prior stroke and patients with AIS was determined using the modified Rankin Scale (MRS). MRS score of prior stroke patients before the onset of COVID-19 was obtained from the electronic medical record system or by follow-up on telephone. For AIS patients with or without COVID-19, MRS score was obtained at discharge.

### Propensity Scores Match Methods

The propensity score match (PSM) method was used to reduce the confounding factor among patients with stroke at baseline. The propensity scores were analyzed using a logistic regression model. The baseline matching variables included age, gender, smoking status, blood pressure, alcohol consumption, and complications, such as diabetes, hypertension, hyperlipidemia, heart disease, cancer, chronic obstructive pulmonary disease (COPD), tuberculosis, chronic kidney disease, liver disease, intracerebral hemorrhage, and asthma. The PSM was established by a nearest-neighbor strategy with ratio 2:1, and caliper 0.02 (12). After matching, a standardized difference was generated and the value <0.1 was taken as an indication of the covariates which were well balanced between the two groups (Supplementary Table S1). Blood pressure at admission was missing in 15.3% of patients, a multivariate imputation by chained equations (MICE) was used to impute SBP and diastolic blood pressure (DBP) (13).

### Statistical Analysis

Continuous variables with non-normal distributions were described as median [interquartile range, IQR]. Categorical variables were described as number and percentage (%). Comparison between two groups was performed with Wilcoxon test for nonparametric variables, Fisher's exact test or chi-square test was performed for categorical variables. Multivariate logistic regression models were developed to explore the relative contribution of risk factors. Variables with *p* < 0.1 in univariate regression were included in multiple regression. No significant interaction was found between variables enrolled in multivariate analyses. The *p* < 0.05 was considered statistically significant. Data preprocessing and statistical analysis were calculated with Ubuntu 16.04.6 and a Rstudio server (R-4.0.3 R Foundation for Statistical Computing).

## Results

### Baseline Characteristics and Outcomes of Patients With COVID-19 and Patients With or Without Prior Stroke

In total, 40,206 patients were found positive for COVID-19 and received in-hospital treatment in Wuhan Province. After excluding patients with missing critical data, the final cohort consisted of 36,358 patients, of whom 1,160 of the patients (3.19%) had prior stroke (Table 1). Patients with prior stroke were significantly older, most of them were male patients (55.4 vs. 48.4%, *p* < 0.001), and had higher SBP (130.00 [114.00, 143.00] vs. 125.00 [114.00, 138.00], *p* < 0.001), with a greater prevalence of smoking (5.1 vs. 2.1%, *p* < 0.001) and alcohol consumption (10.6 vs. 7.3%, *p* < 0.001). COVID-19 patients with prior stroke had more comorbidities, such as hypertension (62.3 vs. 22.1%, *p* < 0.001), diabetes (25.7 vs. 10.3%, *p* < 0.001), hyperlipidemia (5.3 vs. 1.1%, *p* < 0.001), heart disease (23 vs. 6.2%, *p* < 0.001), COPD (9.1 vs. 2.1%, *p* < 0.001), tuberculosis (3 vs. 1.3%, *p* < 0.001), chronic kidney disease (6.6 vs. 2.4%, *p* < 0.001), and cerebral hemorrhage (4 vs. 0.4%, *p* < 0.001). Compared with patients without prior stroke, patients with a positive history had significant leukocytosis, increased neutrophils, lymphopenia, prothrombin time and D-dimer, lower platelet count, hemoglobin, albumin, and alanine aminotransferase (Table 1).

**Table 1:** Baseline characteristics and outcomes of patients with coronavirus disease 2019 (COVID-19) and patient with or without prior stroke.

**Characteristic**	**Overall COVID-19 patient**	**COVID-19 patient without history of stroke**	**COVID-19 patient with history of stroke**	***P*-value**
n	36,358	35,198	1,160	
Age ranges				
<40	6,106 (16.8)	6,098 (17.3)	8 (0.7)	<0.001
40–60	12,950 (35.6)	12,814 (36.4)	136 (11.7)	
60–80	14,805 (40.7)	14,137 (40.2)	668 (57.6)	
>80	2,497 (6.9)	2,149 (6.1)	348 (30.0)	
Sex				
Female	18,696 (51.4)	18,179 (51.6)	517 (44.6)	<0.001
Male	17,662 (48.6)	17,019 (48.4)	643 (55.4)	
Systolic pressure	126.00 [114.00, 138.00]	125.00 [114.00, 138.00]	130.00 [114.00, 143.00]	<0.001
Diastolic pressure	80.00 [72.00, 90.00]	80.00 [73.00, 90.00]	80.00 [71.00, 91.25]	0.472
Smoking	794 (2.2%)	735 (2.1%)	59 (5.1%)	<0.001
Drinking	2,697 (7.4)	2,574 (7.3)	123 (10.6)	<0.001
Diabetes	3,931 (10.8)	3,633 (10.3)	298 (25.7)	<0.001
Hypertension	8,498 (23.4)	7,775 (22.1)	723 (62.3)	<0.001
Hyperlipidemia	459 (1.3)	397 (1.1)	62 (5.3)	<0.001
Heart disease	2,452 (6.7)	2,185 (6.2)	267 (23.0)	<0.001
Cancer	824 (2.3)	799 (2.3)	25 (2.2)	0.796
COPD	850 (2.3)	744 (2.1)	106 (9.1)	<0.001
Tuberculosis	506 (1.4)	471 (1.3)	35 (3.0)	<0.001
Chronic kidney disease	924 (2.5)	847 (2.4)	77 (6.6)	<0.001
Liver disease	1,189 (3.3)	1,138 (3.2)	51 (4.4)	0.028
Intracerebral hemorrhage	178 (0.5)	132 (0.4)	46 (4.0)	<0.001
Asthma	386 (1.1)	374 (1.1)	12 (1.0)	0.927
History of stroke	1,160 (3.2)	0 (0.0)	1,160 (100.0)	<0.001
Disturbance of consciousness	404 (1.1)	339 (1.0)	65 (5.6)	<0.001
White blood cells, × 109 cells/L	5.60 [4.40, 7.10]	5.57 [4.40, 7.06]	6.00 [4.70, 8.14]	<0.001
Neutrophils, × 109 cells/L	3.51 [2.59, 4.90]	3.50 [2.58, 4.86]	4.16 [2.99, 6.03]	<0.001
Lymphocytes, × 109 cells/L	1.30 [0.88, 1.77]	1.31 [0.89, 1.77]	1.09 [0.73, 1.54]	<0.001
Eosnophils, × 109 cells/L	0.06 [0.01, 0.13]	0.07 [0.01, 0.13]	0.06 [0.01, 0.13]	0.083
Monocyte, × 109 cells/L	0.41 [0.30, 0.53]	0.41 [0.30, 0.53]	0.42 [0.31, 0.57]	0.095
Platelets, × 109 cells/L	212.00 [166.00, 267.00]	212.60 [166.00, 267.00]	203.00 [153.00, 257.00]	<0.001
Hemoglobin, g/L	126.00 [115.00, 138.00]	127.00 [116.00, 138.00]	119.00 [106.00, 132.30]	<0.001
Albumin, g/L	38.00 [34.20, 41.40]	38.10 [34.30, 41.50]	35.60 [31.80, 39.20]	<0.001
Alanine aminotransferase, U/L	23.00 [14.90, 38.00]	23.00 [15.00, 38.00]	19.55 [12.83, 31.92]	<0.001
Prothrombin time, s	12.50 [11.50, 13.64]	12.50 [11.50, 13.60]	12.60 [11.60, 13.90]	0.043
D-dimer, μg/L	0.49 [0.23, 1.11]	0.48 [0.22, 1.07]	0.91 [0.45, 2.17]	<0.001
Duration of hospital stay, days	15.00 [9.00, 22.00]	15.00 [9.00, 22.00]	15.00 [9.00, 23.00]	0.440
Non-invasive mechanical ventilation	1,188 (3.3)	1,111 (3.2)	77 (6.6)	<0.001
Invasive mechanical ventilation	818 (2.2)	772 (2.2)	46 (4.0)	<0.001
Disease severity of COVID-19				
Mild	11,921 (32.8)	11,641 (33.1)	280 (24.1)	<0.001
Moderate	16,823 (46.3)	16,299 (46.3)	524 (45.2)	
Severe	6,247 (17.2)	5,967 (17.0)	280 (24.1)	
Critical	1,367 (3.8)	1,291 (3.7)	76 (6.6)	
Died during hospitalization	2,492 (6.9)	2,290 (6.5)	202 (17.4)	<0.001

*COPD, chronic obstructive pulmonary disease; COVID-19, coronavirus disease 2019; Data are reported as number and percentage (%) for categorical variables and median (interquartile range, IQR) for continuous variables.*.

Prior stroke did not increase the length of hospital stay but did increase the requirement for mechanical ventilation (4 vs. 2.4%, *p* < 0.001). In the cohort of patients with prior stroke, 356 (30.7%) patients were diagnosed as being severely and critically ill with COVID-19, and 202 (17.4%) died during their hospitalization. The mortality was 11.6% in the general and mild patients with COVID-19, and 30.6% in the severe and critical patients with COVID-19. Patients with prior history had a higher proportion of severe and critical COVID-19 illness (24.1 and 6.6% vs. 17 and 3.7%, *p* < 0.001), and were significantly more likely to die during their hospitalization (17.4 vs 6.5%, *p* < 0.001), compared with patients without COVID-19 (Table 1).

Propensity-matched analysis was used to adjust for differences in available baseline characteristics and comorbidities. The results showed that 2,170 suitable propensity matches were found for 1,106 (95.3%) of 1,160 COVID-19 patients with prior stroke. After propensity matching, patients with prior stroke still had a higher proportion of severe and critical COVID-19 illness (22.4 and 5.8% vs. 24.2 and 5.8%, *p* = 0.042), and had significantly higher mortality during their hospitalization (16.5 vs. 12.5%, *p* = 0.002) (Supplementary Table S2).

To investigate the effect of MRS scores after IS on the prognosis of COVID-19, we compared the outcomes of patients with COVID-19 in different MRS scores group. We found that patients in MRS score 2–3 and 4–5 have a much higher proportion of severe illness (38.6 and 32.7 vs. 26.5%) and a much higher mortality (20.1 and 26.8 vs. 10.8%) (Supplementary Table S3).

### Analysis of Risk Factors for the Clinical Outcome of Patients With Prior Stroke

**Figure 2:**
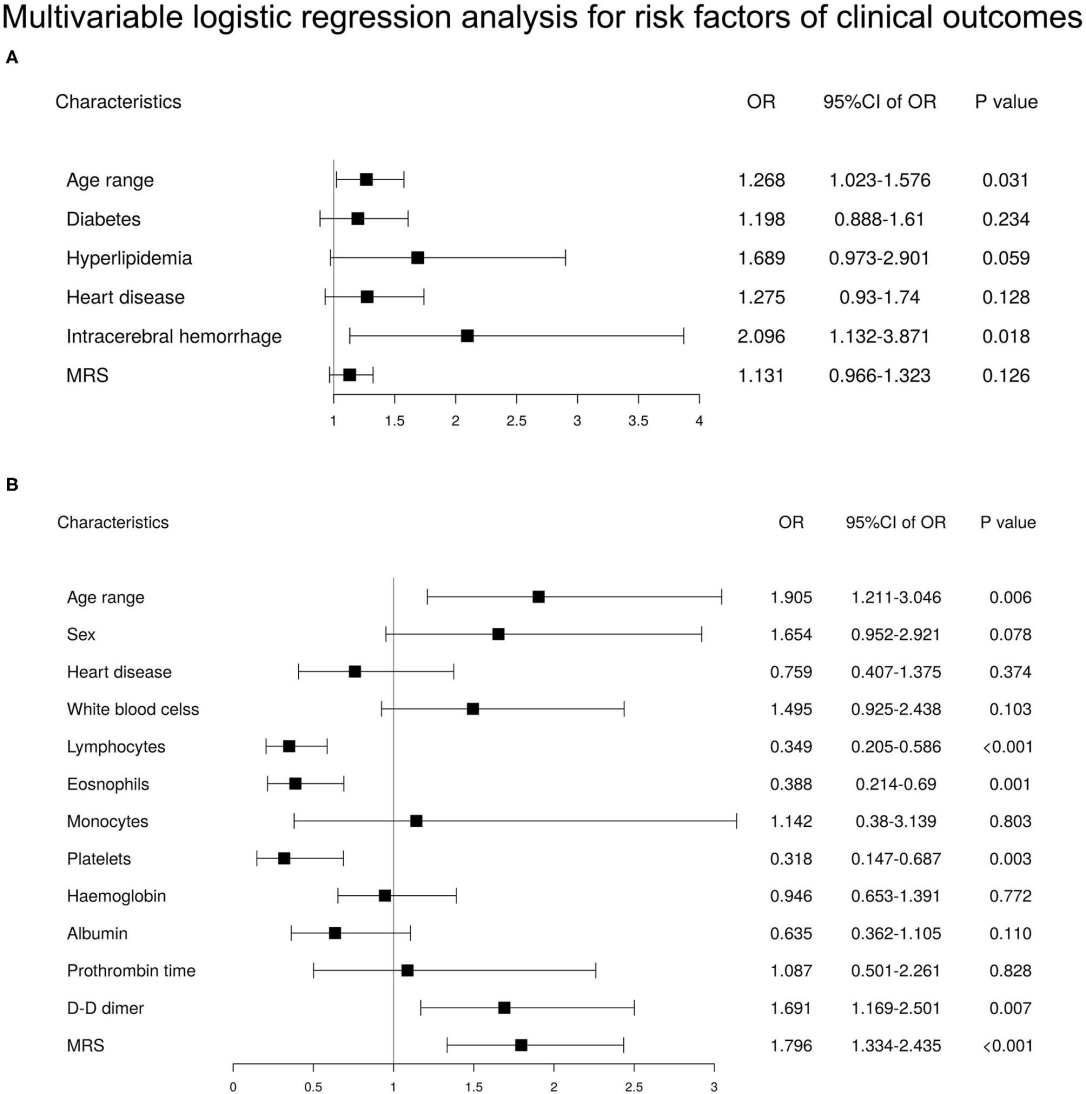
Multivariable logistic regression analysis for risk factors of clinical outcomes in patients with a history of stroke (*n* = 1,160). **(A)** Multivariable logistic regression analysis for risk factors of severe or critical illness in patients with a history of stroke. **(B)** Multivariable logistic regression analysis for risk factors of death in patients with a history of stroke.

To investigate the risk factors for the development of COVID-19 with severe and critical illness and death in patients with prior stroke, we performed a bilateral logistic regression analysis. A univariable logistic regression analysis showed that greater age, hyperlipidemia, heart disease, cerebral hemorrhage, and premorbid MRS grouping were associated with the severe and critical COVID-19 illness (Supplementary Figure 1). Multivariable regression analysis which adjusted for above factors showed that greater age (odds ratio [*OR*] = 1.268 [95% *CI* 1.023–1.576], *p* = 0.031) and cerebral hemorrhage (*OR* = 2.096 [95% *CI* 1.132–3.871], *p* = 0.018) were independent risk factors for the severe and critical COVID-19 illness (Figure 2A).

Higher odds of death were associated with greater age (*OR* = 1.905 [95% *CI* 1.211–3.046], *p* = 0.006), premorbid MRS grouping (*OR* = 1.796 [95% *CI* 1.334–2.435], *p* < 0.001), and laboratory test indices, such as lymphopenia (*OR* = 0.349 [95% *CI* 0.205–0.586], *p* < 0.001), eosinophilopenia (*OR* = 0.388 [95% *CI* 0.214–0.69], *p* = 0.001), and lower platelet count (*OR* = 0.318 [95% *CI* 0.147–0.687], *p* = 0.003) in multivariable regression analysis (Figure 2B). There was no interaction effect between the selected variables.

### The Clinical Characteristics and Outcomes of Patients With AIS

Among a total of 36,358 patients, we identified 124 cases of AIS during their hospitalization, for an incidence of 0.342%. Patients with severe or critical COVID-19 were more likely to have an AIS compared with non-severe groups (0.62 and 0.88% vs. 0.11 and 0.37%, *p* < 0.001), and those with prior stroke had a much higher incidence of AIS (3.36%), with an incidence of 6.58% in the critically ill group (*p* < 0.001) (Table 2).

**Table 2:** Incidence of acute ischemic stroke (AIS) in different disease severity of COVID-19.

**Disease severity of COVID-19**	**Incidence of AIS in** **total COVID-19 patients**	***P*-value**	**Incidence of AIS in COVID-19** **patients with prior ischemic stroke**	***P*-value**
Mild	13/11,921 (0.11)	<0.001	3/280 (1.07)	<0.001
Moderate	60/16,823 (0.37)		16/624 (2.56)	
Severe	39/6,247 (0.62)		15/280 (5.36)	
Critical	12/1,367 (0.88)		5/76 (6.58)	
Total	124/36,358 (0.342)		39/1,160 (3.36)	

*AIS, acute ischemic stroke; COVID-19, coronavirus disease 2019; Data are reported as number and percentage (%) for categorical variables*.

Compared with COVID-19 patients without AIS, the patients with AIS were older (*p* < 0.001) and had higher SBP (131.50 [115.75, 145.75] vs. 126.00 [114.00, 138.00], *p* = 0.013), male gender (58.1 vs. 48.5%, *p* = 0.034), greater prevalence of smoking (7.3 vs. 2.2%, *p* < 0.001), and had a greater rate of comorbidities, such as hypertension (53.2 vs. 23.3%, *p* < 0.001), diabetes (30.6 vs. 10.7%, *p* < 0.001), hyperlipidemia (4.0 vs. 1.3%, *p* = 0.006), heart disease (25 vs. 6.7%, *p* < 0.001), COPD (5.6 vs. 2.3%, *p* = 0.015), chronic kidney disease (6.5 vs. 2.5%, *p* = 0.006), intracerebral hemorrhage (4.0 vs. 0.5%, *p* < 0.001), and prior IS (31.5 vs. 3.1%, *p* < 0.001). In terms of clinical outcomes, COVID-19 patients with AIS had a significantly higher mortality rate (27.4 vs. 6.8%, *p* < 0.001) (Table 3).

**Table 3:** Clinical characteristics of COVID-19 patients with and without AIS.

**Characteristic**	**Overall**	**COVID-19 patients without** **acute ischemic stroke**	**COVID-19 patients with** **acute ischemic stroke**	***P*-value**
N	36,358	36,234	124	
Age ranges				
<40	6,106 (16.8)	6,105 (16.8)	1 (0.8)	<0.001
40–60	12,950 (35.6)	12,933 (35.7)	17 (13.7)	
60–80	14,805 (40.7)	14,744 (40.7)	61 (49.2)	
>80	2,497 (6.9)	2,452 (6.8)	45 (36.3)	
Sex				
Female	18,696 (51.4)	18,644 (51.5)	52 (41.9)	0.034
Male	17,662 (48.6)	17,590 (48.5)	72 (58.1)	
Systolic pressure	126.00 [114.00, 138.00]	126.00 [114.00, 138.00]	131.50 [115.75, 145.75]	0.013
Diastolic pressure	80.00 [72.00, 90.00]	80.00 [72.00, 90.00]	80.50 [70.00, 96.00]	0.630
Smoking	794 (2.2)	785 (2.2)	9 (7.3)	<0.001
Drinking	2,697 (7.4)	2,686 (7.4)	11 (8.9)	0.536
Diabetes	3,931 (10.8)	3,893 (10.7)	38 (30.6)	<0.001
Hypertension	8,498 (23.4)	8,432 (23.3)	66 (53.2)	<0.001
Hyperlipidemia	459 (1.3)	454 (1.3)	5 (4.0)	0.006
Heart disease	2,452 (6.7)	2,421 (6.7)	31 (25.0)	<0.001
Cancer	824 (2.3)	820 (2.3)	4 (3.2)	0.472
COPD	850 (2.3)	843 (2.3)	7 (5.6)	0.015
Tuberculosis	506 (1.4)	502 (1.4)	4 (3.2)	0.081
Chronic kidney disease	924 (2.5)	916 (2.5)	8 (6.5)	0.006
Liver disease	1,189 (3.3)	1,184 (3.3)	5 (4.0)	0.633
Intracerebral hemorrhage	178 (0.5)	173 (0.5)	5 (4.0)	<0.001
Asthma	386 (1.1)	385 (1.1)	1 (0.8)	0.781
History of stroke	1,160 (3.2)	1,121 (3.1)	39 (31.5)	<0.001
White blood cells, × 10^9^ cells/L	5.60 [4.40, 7.10]	5.59 [4.40, 7.10]	7.04 [5.50, 9.31]	<0.001
Neutrophils, × 10^9^ cells/L	3.51 [2.59, 4.90]	3.51 [2.59, 4.89]	4.98 [3.51, 7.42]	<0.001
Lymphocytes, × 10^9^ cells/L	1.30 [0.88, 1.77]	1.30 [0.88, 1.77]	1.17 [0.78, 1.53]	0.020
Eosnophils, × 10^9^ cells/L	0.06 [0.01, 0.13]	0.06 [0.01, 0.13]	0.06 [0.02, 0.13]	0.569
Monocytes, × 10^9^ cells/L	0.41 [0.30, 0.53]	0.41 [0.30, 0.53]	0.49 [0.37, 0.63]	<0.001
Platelets, × 10^9^ cells/L	212.00 [166.00, 267.00]	212.00 [166.00, 267.00]	199.00 [146.75, 248.00]	0.057
Hemoglobin, g/L	126.00 [115.00, 138.00]	126.00 [115.00, 138.00]	125.50 [111.00, 137.25]	0.562
Albumin, g/L	38.00 [34.20, 41.40]	38.00 [34.20, 41.40]	36.50 [32.90, 40.38]	0.017
Alanine aminotransferase, U/L	23.00 [14.90, 38.00]	23.00 [14.90, 38.00]	18.00 [11.93, 27.75]	0.002
Prothrombin time, s	12.50 [11.50, 13.64]	12.50 [11.50, 13.64]	12.15 [11.17, 13.10]	0.021
D-dimer, μg/L	0.49 [0.23, 1.11]	0.49 [0.23, 1.10]	0.95 [0.43, 2.12]	<0.001
Duration of hospital stay, days	15.00 [9.00, 22.00]	15.00 [9.00, 22.00]	13.00 [7.00, 21.25]	0.134
Non-invasive mechanical ventilation	1,188 (3.3)	1,176 (3.2)	12 (9.7)	<0.001
Invasive mechanical ventilation	818 (2.2)	810 (2.2)	8 (6.5)	0.002
Disease severity of COVID-19				
Mild	11,921 (32.8)	11,908 (32.9)	13 (10.5)	<0.001
Moderate	16,823 (46.3)	16,763 (46.3)	60 (48.4)	
Severe	6,247 (17.2)	6,208 (17.1)	39 (31.5)	
Critical	1,367 (3.8)	1,355 (3.7)	12 (9.7)	
Died during hospitalization	2,492 (6.9)	2,458 (6.8)	34 (27.4)	<0.001

*COPD, chronic obstructive pulmonary disease; COVID-19, coronavirus disease 2019; Data are reported as number and percentage (%) for categorical variables and median (IQR) for continuous variables*.

### Analysis of Risk Factors for AIS in Patients With COVID-19

**Figure 3:**
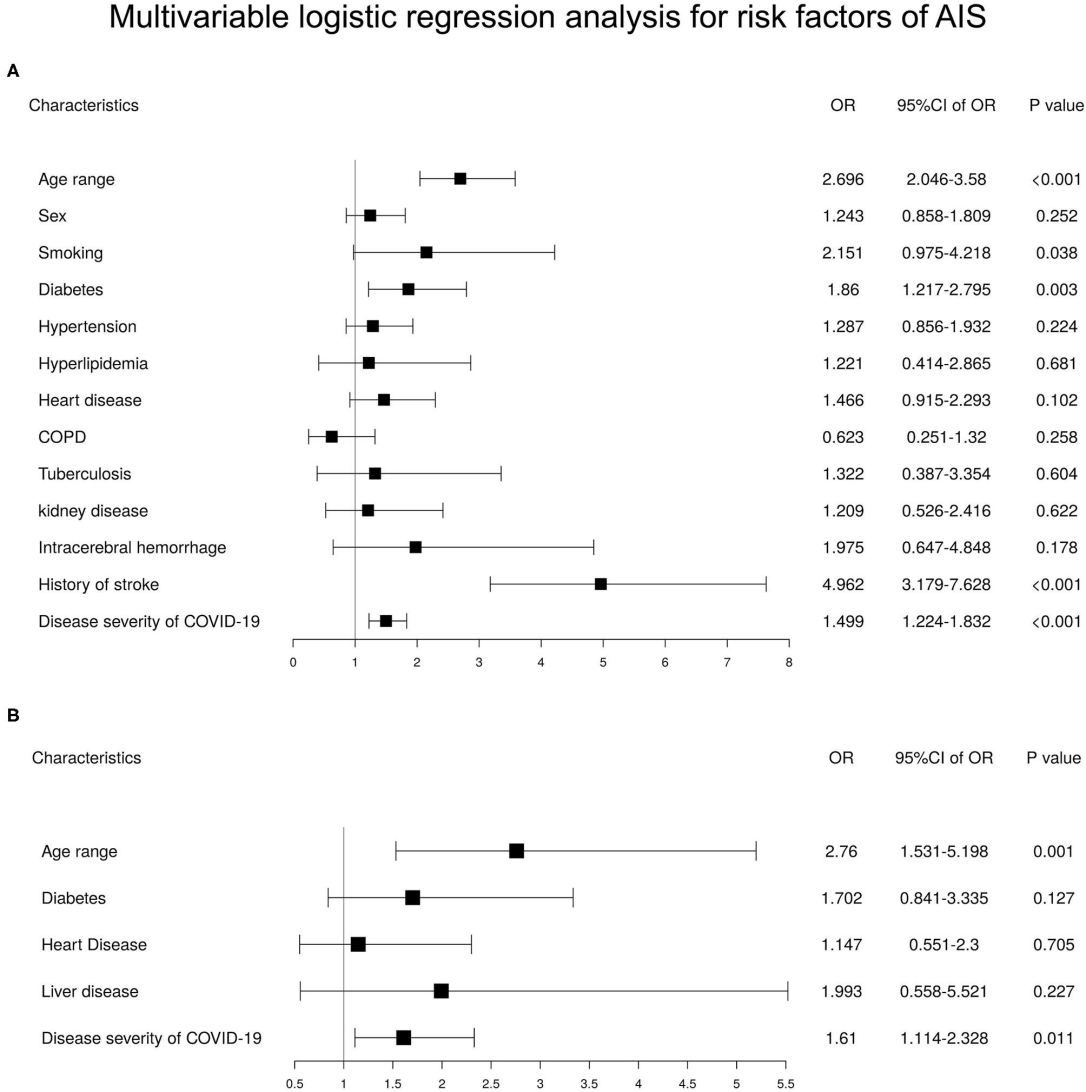
Multivariable logistic regression analysis for risk factors of acute ischemic stroke (AIS) in patients with coronavirus disease 2019 (COVID-19) and COVID-19 patients with a history of stroke. **(A)** Multivariable logistic regression analysis for risk factors of AIS in patients with COVID-19 (*n* = 36,358). **(B)** Multivariable logistic regression analysis for risk factors of AIS in COVID-19 patients with a history of stroke (*n* = 1,160).

In a univariable logistic regression analysis on all 36,358 patients with COVID-19 to study risk factors for AIS, we found greater age, male gender, smoking, hypertension, diabetes, hyperlipidemia, heart disease, COPD, kidney disease, intracerebral hemorrhage, history of stroke, and disease severity of COVID-19 were associated with AIS (Supplementary Figure 2A). A multivariable regression analysis adjusted for factors in the univariable logistic regression analysis revealed that higher odds of AIS were associated with greater age (*OR* = 2.696 [95% *CI* 2.046–3.58], *p* < 0.001), smoking (*OR* = 2.151 [95% *CI* 0.975–4.218], *p* = 0.038), diabetes (*OR* = 1.86 [95% *CI* 1.217–2.795], *p* = 0.003), prior IS (*OR* = 4.962 [95% *CI* 3.179–7.628], *p* < 0.001) and disease severity of COVID-19 (*OR* = 1.499 [95% *CI* 1.224–1.832], *p* < 0.001) (Figure 3A).

In the cohort of 1,160 COVID-19 patients with prior IS, a univariable logistic regression analysis showed only greater age and disease severity of COVID-19 to be associated with AIS (Supplementary Figure 2B). Furthermore, multivariable regression analysis indicated greater age (*OR* = 2.76 [95% *CI* 1.531–5.198], *p* < 0.001) and disease severity of COVID-19 (*OR* = 1.61 [95% *CI* 1.114–2.328], *p* = 0.011) to be independent risk factors (Figure 3B).

## Discussions

In this study, we included the whole dataset of hospitalized patients with COVID-19 in Wuhan city. By mid-April 2020, when the first wave of COVID-19 was over, a total of 50,333 patients were diagnosed with COVID-19, in which 40,206 had been hospitalized. This unique cohort may facilitate to draw more reliable conclusions regarding the relationship between IS and COVID-19. We found COVID-19 patients with prior stroke experienced worse clinical outcomes than those without. After propensity matching for age, sex, and all significant co-morbidities, history of stroke retained its strong association with the severity of disease and death from COVID-19. Our results suggested that prior stroke may have an adverse effect on the prognosis of COVID-19. In addition to other reported risk factors, we found that the risk of death from COVID-19 in patients with prior stroke were associated with premorbid MRS grouping for the first time. MRS is a widely used scale that has been proven to be a valid and reliable instrument for defining the impairment of neurological function in patients with stroke (14). In our patients with MRS scores of 4–5, the mortality was as high as 26.8%. Patients with more severe neurological deficit experienced worse clinical outcomes after the infection may be due to impaired mechanism for delivery secretions in respiratory tract and host immune responses (6, 15). Our results indicated that the hospitalized COVID-19 patients with prior stroke, and particularly those with high MRS scores, have a markedly increased risk of death, such that these vulnerable patients need very close monitoring and aggressive treatment measures.

While the exact relationship between AIS and COVID-19 is still not fully clarified (16, 17), in the early days of the epidemic, some small sample studies found COVID-19 infection is significantly associated with AIS compared with control subjects with an OR of 3.9 (18), and later evidence indicated that the infection can trigger IS or worsen an existing stroke (19, 20). In this study, we reported a stroke incidence of 0.342% in Wuhan city, which corresponds to an annual incidence of 345/100,000 person-years for IS (21) within an average course of ~20 days following admission, and was higher than the in-hospital stroke rate of 0.11% (22). The stroke incidence we reported was lower than that in some previous reports. Other studies from heavily affected regions have suggested a stroke risk among patients hospitalized with COVID-19 in the range of 0.5–5% (23–25), with the most recent single center, multi-center studies or meta-analysis reporting a stroke rate of 1–2% among hospitalized patients (26, 27). However, the stroke incidence in this study was comparable with that in a multinational study, which reported an overall stroke risk of 0.5 and 0.3% in Asia (11). In addition, we note that the recurrence of IS is a particular problem which has not been examined in previous studies in the context of the COVID-19 pandemic. In this study, we found that patients with COVID-19 with prior stroke had a 3.36% risk of AIS, which is compared with a 5% annual recurrence rate for patients with stroke in the general population (28), it indicated that a patient with COVID-19 during infection has a much higher rate of AIS. The disease severity of COVID-19 was suspected to associate with the occurrence of AIS (29, 30), since AIS has been reported to occur more frequently in severe ill patients (31). In this study, we first found that the occurrence of AIS was independently associated with the greater disease severity of COVID-19 in the patients after adjusting for age, sex, and comorbidities. The independent association between AIS and the disease severity of COVID-19 may further explain why the stroke incidence rate varies greatly across different regions, centers, and levels of hospitals, since some centers have relaxed criteria for admission, while the other centers with excessive disease burden have adopted strict criteria. Other possible reasons for differences between centers include ethnicity differences, small sample size, and publication bias. As a whole region study, we have included all inpatients in Wuhan, which should have reduced the influence of differing admission criteria between hospitals. However, the rate of AIS in our study may be underestimated, as the detection of AIS symptoms is challenging in those critically ill with COVID-19, especially those typically intubated and sedated. In addition, our study suggests that when reporting AIS caused by COVID-19, the severity or diagnostic criteria of COVID-19 in patients enrolled should be specified, or there may be a lack of comparability between groups of data.

To get a better understanding of the risk factors and clinical outcomes of patients with AIS following COVID-19, we selected AIS patients without COVID-19 who were hospitalized in Tongji Hospital over the same period as a control group. We found that AIS among patients with COVID-19 occurred more commonly in elderly patients, and showed a lesser association with hypertension, hyperlipidemia, smoking, and alcohol consumption, but more association with comorbidities, such as heart disease, COPD, kidney disease, liver disease, and prior IS (Supplementary Table S4). These differences in demographic characteristics and risk factors suggested that AIS occurred following the COVID-19 hospitalization may differ in its etiology from conventional stroke. Unfortunately, we failed to do TOAST classification due to the incompleteness of examinations during the epidemic. We did not study the mechanisms of stroke following COVID-19, the key proposed mechanisms include the development of cytokine storm and activation of the innate immune system, embolic events propagated by pre-existing or new-onset arrhythmias, hypoxia-induced ischemia secondary to severe pulmonary disease, thrombotic microangiopathy, endotheliopathy/endothelialitis, and multifactorial activation of coagulation (32). AIS patients with COVID-19 had a higher ratio of MRS 4–5 at discharge, and a much higher mortality, this is consistent with previous studies (19, 33). Given that COVID-19 is prone to coagulation disorders, such as pulmonary embolism and venous thrombosis (34), patients at high risk might benefit from an anticoagulant or antiplatelet therapy, but further research is needed.

A notable strength of our study is that we included all patients with COVID-19 from hospitals across Wuhan State, such that our results should be more representative than existing studies from a single hospital system or multi-centers (24, 25). We thus provided a more reliable assessment of the incidence of AIS after COVID-19 and illustrated its correlation with the severity of COVID-19. Although there are many multi-center studies and meta-analyses on the incidence of AIS in patients with COVID-19, but the majority of studies included are either case series or case reports, with only a few being observational cohort studies, thus, many of these studies are considered relatively lower in the quality with publication and reporting bias, and stratification of patients' AIS risk based on the COVID-19 severity may be of more interest to clinicians. To the best of our knowledge, this is the largest retrospective cohort study among COVID-19 patients with prior stroke who have experienced a definite outcome, which, as such, enabled us to have identified severe illness and other mortality associated risk factors, notably premorbid MRS in those patients with prior stroke.

## Limitations

The main limitation of this study arises from the retrospective study design, and the necessity of identifying the primary exposure and outcome, as well as confounding factors, using hospital electronic records from administrative datasets, which may vary in assessment criteria between hospitals. Second, this study took place in a severely challenged health system during the local peak of the COVID-19 pandemic, such that some reports or test results were simply unavailable, which could have introduced reporting or indication biases. In addition, we cannot address the possibility that treatment protocols may have varied based on local experience.

## Conclusions

The major finding of our regional study is that the prior stroke is associated with an increased risk of developing severe course of COVID-19 and death. In patients hospitalized with COVID-19 with prior stroke, with high premorbid MRS scores have a markedly increased risk of death. COVID-19 increased the incidence of AIS, especially in patients with a prior IS, and the incidence is independently correlated with the severity of COVID-19. Nevertheless, these results will hopefully provide guidance for clinicians to understand the interactions between COVID-19 and IS, and are conducive to the management of patients with prior stroke who are at a high risk for COVID-19.

## Data Availability Statement

The raw data supporting the conclusions of this article will be made available by the authors, without undue reservation.

## Ethics Statement

This study was designed by the authors and approved with provision of a waiver of authorization and informed consent by the Ethics Committee of Tongji Hospital (IRB ID: TJ-C20200121).

## Author Contributions

MW, HZ, GL, SX, and WW: concept and design. MW, HZ, YH, CQ, XinL, and YuT: acquisition, analysis, and interpretation of data. MW, HZ, SX, and WW: drafting of the manuscript and critical revision of the manuscript for important intellectual content. ML, YuT, XiaL, and GY: statistical analysis. SX and WW: obtained funding. YH, CQ, XinL, and YiT: administrative, technical, and material support. SX and WW: supervision.

## Funding

This study was funded by a coronavirus disease 2019 (COVID-19) Emergency Project from Wuhan Science and Technology Bureau (2020020401010096) and the Ministry of Science and Technology of People's Republic of China (2020YFC0841301).

## Conflict of Interest

ML, YuT, XL, and GY are employed by Winning Health Technology Group Co. Ltd. The remaining authors declare that the research was conducted in the absence of any commercial or financial relationships that could be construed as a potential conflict of interest.

## Publisher's Note

All claims expressed in this article are solely those of the authors and do not necessarily represent those of their affiliated organizations, or those of the publisher, the editors and the reviewers. Any product that may be evaluated in this article, or claim that may be made by its manufacturer, is not guaranteed or endorsed by the publisher.

## References

[1] Johnson CO Nguyen M Roth GA Nichols E Alam T Abate D. Global, regional, and national burden of stroke, 1990–2016: a systematic analysis for the global burden of disease study 2016. Lancet Neurol. (2019) 18:439–58. 10.1016/S1474-4422(19)30034-1 30871944 PMC6494974

[2] Lavados PM Hoffmeister L Moraga AM Vejar A Vidal C Gajardo C. Incidence, risk factors, prognosis, and health-related quality of life after stroke in a low-resource community in chile (nandu): a prospective population-based study. Lancet Glob Health. (2021) 9:e340–e351. 10.1016/S2214-109X(20)30470-8 33422189

[3] Qin C Zhou L Hu Z Yang S Zhang S Chen M. Clinical characteristics and outcomes of covid-19 patients with a history of stroke in wuhan, china. Stroke. (2020) 51:2219–23. 10.1161/STROKEAHA.120.030365 32466735 PMC7282412

[4] Nalleballe K Siddamreddy S Sheng S Dandu V Arulprakash N Kovvuru S. Coronavirus disease 2019 in patients with prior ischemic stroke. Cureus. (2020) 12:e10231. 10.7759/cureus.10231 33042672 PMC7535872

[5] Perry RJ Smith CJ Roffe C Simister R Narayanamoorthi S Marigold R. Characteristics and outcomes of covid-19 associated stroke: a uk multicentre case-control study. J Neurol Neurosurg Psychiatry. (2020) 92:242–8. 10.1136/jnnp-2020-324927 33154179

[6] Elkind MSV Boehme AK Smith CJ Meisel A Buckwalter MS. Infection as a stroke risk factor and determinant of outcome after stroke. Stroke. (2020) 51:3156–68. 10.1161/STROKEAHA.120.030429 32897811 PMC7530056

[7] Pun BT Badenes R Heras La Calle G Orun OM Chen W Raman R. Prevalence and risk factors for delirium in critically ill patients with covid-19 (covid-d): a multicentre cohort study. Lancet Respir Med. (2021) 9:239–50. 10.1016/S2213-2600(20)30552-X 33428871 PMC7832119

[8] Grau AJ Urbanek C Palm F. Common infections and the risk of stroke. Nat Rev Neurol. (2010) 6:681–94. 10.1038/nrneurol.2010.163 21060340

[9] Bhatia R Pedapati R Komakula S Srivastava MVP Vishnubhatla S Khurana D. Stroke in coronavirus disease 2019: a systematic review. J Stroke. (2020) 22:324–35. 10.5853/jos.2020.02264 33053948 PMC7568983

[10] Fridman S Bres Bullrich M Jimenez-Ruiz A Costantini P Shah P Just C. Stroke risk, phenotypes, and death in covid-19. Neurology. (2020) 95:e3373–85. 10.1212/WNL.0000000000010851 32934172

[11] Shahjouei S Naderi S Li J Khan A Chaudhary D Farahmand G. Risk of stroke in hospitalized sars-cov-2 infected patients: a multinational study. EBioMedicine. (2020) 59:102939. 10.1016/j.ebiom.2020.102939 32818804 PMC7429203

[12] Geldof T Popovic D Van Damme N Huys I Van Dyck W. Nearest neighbour propensity score matching and bootstrapping for estimating binary patient response in oncology: a monte carlo simulation. Sci Rep. (2020) 10:964. 10.1038/s41598-020-57799-w 31969627 PMC6976708

[13] Zhang ZH. Multiple imputation with multivariate imputation by chained equation (mice) package. Ann Transl Med. (2016) 4:30. 10.3978/j.issn.2305-5839.2015.12.63 26889483 PMC4731595

[14] Kasner SE. Clinical interpretation and use of stroke scales. Lancet Neurol. (2006) 5:603–12. 10.1016/S1474-4422(06)70495-1 16781990

[15] Westendorp WF Nederkoorn PJ Vermeij JD Dijkgraaf MG van de Beek D. Post-stroke infection: a systematic review and meta-analysis. BMC Neurol. (2011) 11:110. 10.1186/1471-2377-11-110 21933425 PMC3185266

[16] Spence JD de Freitas GR Pettigrew LC Ay H Liebeskind DS Kase CS. Mechanisms of stroke in covid-19. Cerebrovasc Dis. (2020) 49:451–8. 10.1159/000509581 32690850 PMC7445374

[17] Butt JH Fosbol EL Ostergaard L Yafasova A Andersson C Schou M. Effect of covid-19 on first-time acute stroke and transient ischemic attack admission rates and prognosis in denmark: a nationwide cohort study. Circulation. (2020) 142:1227–9. 10.1161/CIRCULATIONAHA.120.050173 32755320 PMC7497886

[18] Belani P Schefflein J Kihira S Rigney B Delman BN Mahmoudi K. Covid-19 is an independent risk factor for acute ischemic stroke. Am J Neuroradiol. (2020) 41:1361–4. 10.3174/ajnr.A6650 32586968 PMC7658882

[19] Ntaios G Michel P Georgiopoulos G Guo Y Li W Xiong J. Characteristics and outcomes in patients with covid-19 and acute ischemic stroke: The global covid-19 stroke registry. Stroke. (2020) 51:e254–e258. 10.1161/STROKEAHA.120.031208 32787707 PMC7359900

[20] Qureshi AI Abd-Allah F Al-Senani F Aytac E Borhani-Haghighi A Ciccone A. Management of acute ischemic stroke in patients with covid-19 infection: Insights from an international panel. Am J Emerg Med. (2020) 38:1548 e1545–1548 e1547. 10.1016/j.ajem.2020.05.018 32444298 PMC7211609

[21] Wang W Jiang B Sun H Ru X Sun D Wang L. Prevalence, incidence, and mortality of stroke in china. Circulation. (2017) 135:759–71 10.1161/CIRCULATIONAHA.116.025250 28052979

[22] Walkey AJ Wiener RS Ghobrial JM Curtis LH Benjamin EJ. Incident stroke and mortality associated with new-onset atrial fibrillation in patients hospitalized with severe sepsis. JAMA. (2011) 306:2248–54. 10.1001/jama.2011.1615 22081378 PMC3408087

[23] Markus HS Brainin M. Covid-19 and stroke—a global world stroke organization perspective. Int J Stroke. (2020) 15:361–4. 10.1177/1747493020923472 32310017 PMC11927026

[24] Merkler AE Parikh NS Mir S Gupta A Kamel H Lin E. Risk of ischemic stroke in patients with coronavirus disease 2019 (covid-19) vs patients with influenza. JAMA Neurol. (2020) 77:1–7. 10.1001/jamaneurol.2020.2730 32614385 PMC7333175

[25] Modin D Claggett B Sindet-Pedersen C Lassen MCH Skaarup KG Jensen JUS. Acute covid-19 and the incidence of ischemic stroke and acute myocardial infarction. Circulation. (2020) 142:2080–2. 10.1161/CIRCULATIONAHA.120.050809 33054349 PMC7682795

[26] Nannoni S de Groot R Bell S Markus HS. Stroke in covid-19: A systematic review and meta-analysis. Int J Stroke. (2021) 16:137–49. 10.1177/1747493020972922 33103610 PMC7859578

[27] Tan YK Goh C Leow AST Tambyah PA Ang A Yap ES. Covid-19 and ischemic stroke: A systematic review and meta-summary of the literature. J Thromb Thrombolysis. (2020) 50:587–95. 10.1007/s11239-020-02228-y 32661757 PMC7358286

[28] Flach C Muruet W Wolfe CDA Bhalla A Douiri A. Risk and secondary prevention of stroke recurrence: a population-base cohort study. Stroke. (2020) 51:2435–44. 10.1161/STROKEAHA.120.028992 32646337 PMC7382537

[29] Viguier A Delamarre L Duplantier J Olivot JM Bonneville F. Acute ischemic stroke complicating common carotid artery thrombosis during a severe covid-19 infection. J Neuroradiol. (2020) 47:393–4. 10.1016/j.neurad.2020.04.003 32389423 PMC7196531

[30] Keller E Brandi G Winklhofer S Imbach LL Kirschenbaum D Frontzek K. Large and small cerebral vessel involvement in severe covid-19: Detailed clinical workup of a case series. Stroke. (2020) 51:3719–22. 10.1161/STROKEAHA.120.031224 33054673 PMC7678671

[31] Siepmann T Sedghi A Simon E Winzer S Barlinn J de With K. Increased risk of acute stroke among patients with severe covid-19: a multicenter study and meta-analysis. Eur J Neurol. (2021) 28:238–47. 10.1111/ene.14535 32920964

[32] Zakeri A Jadhav AP Sullenger BA Nimjee SM. Ischemic stroke in covid-19-positive patients: an overview of sars-cov-2 and thrombotic mechanisms for the neurointerventionalist. J Neurointerv Surg. (2021) 13:202–6. 10.1136/neurintsurg-2020-016794 33298508

[33] Richter D Krogias C Eyding J Bartig D Grau A Weber R. Comparison of stroke care parameters in acute ischemic stroke patients with and without concurrent covid-19. a nationwide analysis. Neurol Res Pract. (2020) 2:48. 10.1186/s42466-020-00095-9 33230500 PMC7675387

[34] Levi M Thachil J Iba T Levy JH. Coagulation abnormalities and thrombosis in patients with covid-19. Lancet Haematol. (2020) 7:e438–40. 10.1016/S2352-3026(20)30145-9 32407672 PMC7213964

