# Supplementary material for: Association Between Ischemic Stroke and COVID-19 in China: A Population-Based Retrospective Study
Source: Front Med (Lausanne). 2022 Feb 21;8:792487. doi: 10.3389/fmed.2021.792487 (PMC8898959; doi:10.3389/fmed.2021.792487)
Supplement: Supplementary file 1 [file Table_1.DOCX]

**Supplementary Tables**

| Table s1. Baseline characteristics of COVID-19 patients with or without prior stroke and balance diagnostics after propensity scores matching. | | | | | |
| --- | --- | --- | --- | --- | --- |
| Characteristic | Overall | COVID-19 patients without prior stroke | COVID-19 patients with prior stroke | P | Standardized difference |
| N | 3276 | 2170 | 1106 |  |  |
| Age ranges | | | | |  |
| <40 | 24 (0.7) | 16 (0.7) | 8 (0.7) | 0.96 | <0.001 |
| 40-60 | 347 (10.6) | 222 (10.2) | 125 (11.3) |  |  |
| 60-80 | 2048 (62.5) | 1376 (63.4) | 672 (60.8) |  |  |
| >80 | 857 (26.2) | 556 (25.6) | 301 (27.2) |  |  |
| Sex | | | | | |
| Female | 1806 (55.2) | 1203 (55.4) | 603 (54.5) | 0.6 | 0.018 |
| Male | 1470 (44.8) | 967 (44.6) | 503 (45.5) |  |  |
| Systolic pressure | 126 [114, 138] | 125 [114, 138] | 126 [114, 138] | 0.74 | 0.001 |
| Diastolic pressure | 80 [72,90] | 86 [72,90] | 87 [72,90] | 0.97 | 0.012 |
| Smoking | 154 (4.7) | 102 (4.7) | 52 (4.7) | 1 | <0.001 |
| Drinking | 337 (10.3) | 222 (10.2) | 115 (10.3) | 0.929 | 0.006 |
| Diabetes | 777 (23.7) | 513 (23.6) | 264 (23.9) | 0.918 | 0.005 |
| Hypertension | 1988 (60.7) | 1319 (60.7) | 669 (60.4) | 0.900 | 0.006 |
| Hyperlipidemia | 115 (3.5) | 68 (3.1) | 47 (4.2) | 0.123 | 0.059 |
| Heart disease | 633 (19.3) | 400 (18.4) | 233 (21.1) | 0.078 | 0.066 |
| Cancer | 76 (2.2) | 51 (2.3) | 25 (2.2) | 0.969 | 0.006 |
| COPD | 224 (6.8) | 143 (6.6) | 81 (7.3) | 0.475 | 0.029 |
| Tuberculosis | 78 (2.3) | 49 (2.3) | 29 (2.6) | 0.600 | 0.024 |
| Chronic kidney disease | 178 (5.7) | 127 (5.8) | 62 (5.6) | 0.836 | 0.11 |
| Liver disease | 110 (3.4) | 69 (3.2) | 41 (3.7) | 0.490 | 0.029 |
| Intracerebral hemorrhage | 74 (2.3) | 43 (2.0) | 31 (2.8) | 0.1701 | 0.054 |
| Asthma | 35 (1.1) | 23 (1.0) | 12 (1.1) | 1 | 0.002 |

| Table s2. Comparing of premorbid MRS in COVID-19 patients with a prior ischemic stroke with different outcomes | | | | | | | |
| --- | --- | --- | --- | --- | --- | --- | --- |
| Premorbid MRS grouping | Overall | Mild-Moderate | Severe-Critical | P | Alive | Deceased | P |
| 0-1 | 547 (50.7) | 402 (73.5) | 145 (26.5) | 0.002 | 488 (89.2) | 59 (10.8) | <0.001 |
| 2-3 | 259 (24.0) | 159 (61.4) | 100 (38.6) |  | 207 (79.9) | 52 (20.1) |  |
| 4-5 | 272 (25.2) | 183 (67.3) | 89 (32.7) |  | 199 (73.2) | 73 (26.8) |  |
| Abbreviations: COVID-19, coronavirus disease 2019; MRS, modified Rankin Scale; Data are reported as number and percentage (%) for categorical variable. | | | | | | | |

| Table s3.Comparing of premorbid MRS in COVID-19 patients with a prior ischemic stroke with different outcomes | | | | | | | |
| --- | --- | --- | --- | --- | --- | --- | --- |
| Premorbid MRS grouping | Overall | Mild-Moderate | Severe-Critical | P | Alive | Deceased | P |
| 0-1 | 547 | 402 (73.5) | 145 (26.5) | 0.002 | 488 (89.2) | 59 (10.8) | <0.001 |
| 2-3 | 259 | 159 (61.4) | 100 (38.6) |  | 207 (79.9) | 52 (20.1) |  |
| 4-5 | 272 | 183 (67.3) | 89 (32.7) |  | 199 (73.2) | 73 (26.8) |  |
| Abbreviations: COVID-19, coronavirus disease 2019; MRS, modified Rankin Scale;  Data are reported as number and percentage (%) for categorical variable. | | | | | | | |

**Clinical characteristics of AIS cases with and without COVID-19**

To further depict the clinical characteristics of AIS cases with COVID-19, we included a cohort of 226 patients of AIS without COVID-19 who were consecutively admitted to Tongji Hospital during the same period as control to compare risk factors, clinical characteristics and prognosis of AIS patients with and without COVID-19. Compared with uncomplicated AIS patients, the AIS patients with COVID-19 were older, had lower SBP (131.50 [115.75, 145.75] vs 142.00 [130.00, 157.00], p<0.001), significantly lower prevalence of smoking (7.3% vs 35.4%, p<0.001) and alcohol consumption (8.9% vs 28.8%, p<0.001), and lower rate of hyperlipidemia (4.0% vs 11.5%, p=0.019), but a greater rate of comorbidities such as heart disease (25.0% vs 15.5%, p=0.030), COPD (5.6% vs 0%, p<0.001), kidney disease (6.5% vs 1.3%, p=0.009), liver disease (4.0% vs 0.9%, p=0.044), and prior ischemic stroke (31.5% vs 21.2%, p=0.034). Laboratory test showed increased neutrophils, lymphopenia, eosinophil penia, lower platelet count, hemoglobinopenia, higher albumin, increased D-D-dimer, and shortened prothrombin times in AIS patients with COVID-19.

In terms of medication, the COVID-19 group was more likely to receive anticoagulant therapy (20.2% vs 1.8%, p<0.001), while the non-COVID-19 group received more antiplatelet therapy (43.5% vs 88.9%, p<0.001). AIS patients with COVID-19 had a longer hospital stay (13.00 [7.00, 21.25] vs 8.00 [6.00, 11.75], p<0.001), more frequent rating MRS 4 – 5 (44.3 vs 15.6, p<0.001), and a much higher mortality (27.4% vs 2.2%, p<0.001) (Table s4 in the Supplementary).

| Table s4. Comparing of acute ischemic stroke patients with and without COVID-19 | | | |
| --- | --- | --- | --- |
| Characteristic | AIS without COVID-19 | AIS with COVID-19 | p |
| n | 226 | 124 |  |
| Age, y | | | |
| <40 | 7 (3.1) | 1 (0.8) | <0.001 |
| 40-60 | 99 (43.8) | 17 (13.7) |  |
| 60-80 | 111 (49.1) | 61 (49.2) |  |
| >80 | 9 (4.0) | 45 (36.3) |  |
| Sex | | | |
| Female | 82 (36.3) | 52 (41.9) | 0.298 |
| Male | 144 (63.7) | 72 (58.1) |  |
| Systolic pressure | 142.00 [130.00, 157.00] | 131.50 [115.75, 145.75] | <0.001 |
| diastolic pressure | 83.00 [77.00, 93.00] | 80.50 [70.00, 96.00] | 0.301 |
| Smoking | 65 (28.8) | 11 (8.9) | <0.001 |
| Drinking | 80 (35.4) | 9 (7.3) | <0.001 |
| Diabetes | 50 (22.1) | 38 (30.6) | 0.079 |
| Hypertension | 141 (62.4) | 66 (53.2) | 0.095 |
| Hyperlipidemia | 26 (11.5) | 5 (4.0) | 0.019 |
| Heart disease | 35 (15.5) | 31 (25.0) | 0.03 |
| Cancer | 6 (2.7) | 4 (3.2) | 0.759 |
| COPD | 0 (0.0) | 7 (5.6) | <0.001 |
| Tuberculosis | 2 (0.9) | 4 (3.2) | 0.107 |
| Chronic kidney disease | 3 (1.3) | 8 (6.5) | 0.009 |
| Liver disease | 2 (0.9) | 5 (4.0) | 0.044 |
| Intracerebral hemorrhage | 5 (2.2) | 5 (4.0) | 0.328 |
| History of stroke | 48 (21.2) | 39 (31.5) | 0.034 |
| Asthma | 0 (0.0) | 1 (0.8) | 0.176 |
| Antiplatelet | 201 (88.9) | 54 (43.5) | <0.001 |
| Anticoagulant | 4 (1.8) | 25 (20.2) | <0.001 |
| Duration of hospital stay, days | 8.00 [6.00, 11.75] | 13.00 [7.00, 21.25] | <0.001 |
| MRS | | | |
| 0-1 | 105 (46.8) | 22 (25.0) | <0.001 |
| 2-3 | 84 (37.5) | 27 (30.7) |  |
| 4-5 | 35 (15.6) | 39 (44.3) |  |
| Died during hospitalization | 5 (2.2) | 34 (27.4) | <0.001 |

**Supplementary Figures**

Figure 1 in the Supplement. Univariable logistic regression analysis for risk factors of clinical outcomes. (A) Univariable logistic regression analysis for risk factors of severe or critical illness in patients with a history of stroke. (B) Univariable logistic regression analysis for risk factors of death in patients with a history of stroke.


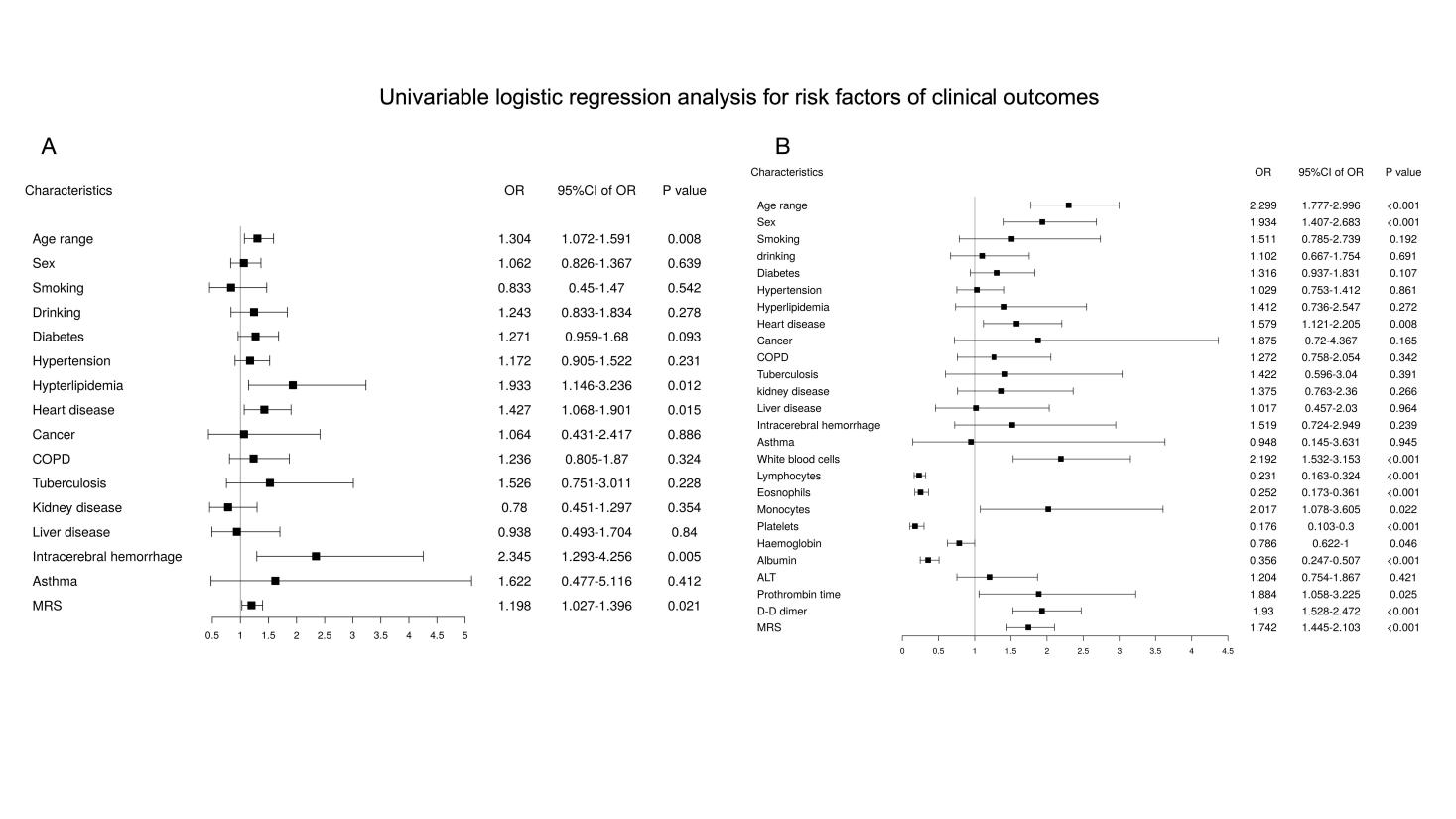


Figure 2 in the Supplement. Univariable logistic regression analysis for risk factors of AIS. (A) Univariable logistic regression analysis for risk factors of AIS in COVID-19 patients. (B) Univariable logistic regression analysis for risk factors of AIS in COVID-19 patients with a history of stroke.


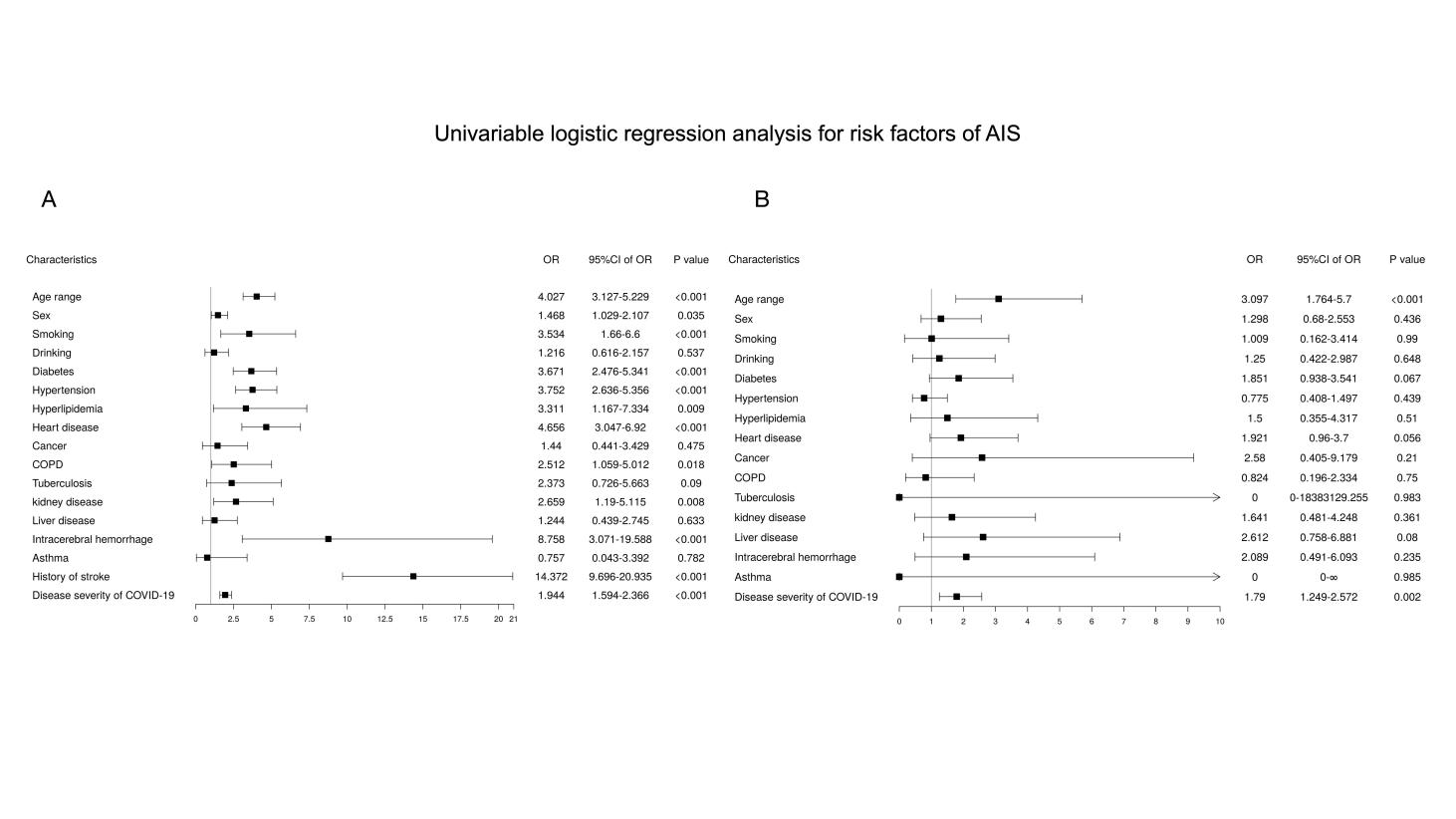


**Supplementary Methods**

Standardized collection and processing method

Electronic Medical Record (EMR) from 61 Wuhan hospitals and mobile cabin hospitals were saved as Comma-Separated Values (CSV) file and collected to primary data node. CSV files from each hospital includes the EMR of all COVID-19 patients from admission to discharge.

EMR from primary data node contained both shareable EMR and non-shareable EMR. A standardized processing method was developed to create a functional and semantic interoperability dataset for further analysis. A metadata directory was created to manage all primary EMR. The metadata directory was built up by mapping,  cleaning and converting the rule metadata. Since each CSV record type contains its own metadata which is not sharable, a rule-based system map metadata from each CSV file was added to the metadata directory. After data cleaning and data quality validation, incomplete and wrong data was removed, rule metadata including function name parameters, data types were converted to a new form.

Using the metadata directory, a common data model is built up to combine all CSV files into a standardized form. Based on HL7 Clinical Document Architecture and national information classification of medical records, a clinical data information model is mapped with standardized clinical metadata. Then an Observational Medical Outcomes Partnership common data model based COVID-19 data model is generated in this study.

During processing, information includes patients name, identity (ID), address and other personal related information are anonymized. Bias data is detected and cleaned. All data are generated with standardized format. Similar data was removed after similarity detection.

After COVID-19 data model is generated, COVID-19 related clinical information are extracted from data model. Key parameters contain baseline information, end status, physical examination, history, clinical symptoms, laboratory test, treatment related information, Computed Tomography (CT) features, non-drug treatment information.

| Disease severity of COVID-19 | |
| --- | --- |
| Mild | Light clinical symptoms and no sign of pneumonia on lung imaging |
| Moderate | Fever, respiratory tract symptoms and other symptoms; Imaging suggests pneumonia |
| Severe | Any of the following: (1) respiratory distress, respiration rate ≥ 30 times / min; (2) oxygen saturation ≤ 93% at rest; (3) PaO2 / FiO2 ≤ 300 mmHg (1mmHg = 0.133 kPa) |
| Critical | As in severe + any of the following: (1) respiratory failure occurs and mechanical ventilation is required; (2) shock; (3) complicated with other organ failure and need of intensive care unit (ICU) monitoring and treatment |
